# Beneficial effects of the novel marine oxygen carrier M101 during cold preservation of rat and human pancreas

**DOI:** 10.1111/jcmm.14666

**Published:** 2019-10-11

**Authors:** Florent Lemaire, Séverine Sigrist, Eric Delpy, Julien Cherfan, Claude Peronet, Franck Zal, Karim Bouzakri, Michel Pinget, Elisa Maillard

**Affiliations:** ^1^ UMR DIATHEC EA 7294 Centre Européen d’Etude du Diabète Université de Strasbourg Strasbourg France; ^2^ HEMARINA Aéropôle Centre Biotechnopôle Morlaix France

**Keywords:** cold ischaemia, islet isolation, islet transplantation, necrosis, oxidative stress, oxygen carrier, pancreas preservation

## Abstract

Ischaemia impairs organ quality during preservation in a time‐dependent manner, due to a lack of oxygen supply. Its impact on pancreas and islet transplantation outcome has been demonstrated by a correlation between cold ischaemia time and poor islet isolation efficiency. Our goal in the present study was to improve pancreas and islet quality using a novel natural oxygen carrier (M101, 2 g/L), which has been proven safe and efficient in other clinical applications, including kidney transplantation, and for several pre‐clinical transplantation models. When M101 was added to the preservation solution of rat pancreas during ischaemia, a decrease in oxidative stress (ROS), necrosis (HMGB1), and cellular stress pathway (p38 MAPK)activity was observed. Freshly isolated islets had improved function when M101 was injected in the pancreas. Additionally, human pancreases exposed to M101 for 3 hours had an increase in complex 1 mitochondrial activity, as well as activation of AKT activity, a cell survival marker. Insulin secretion was also up‐regulated for isolated islets. In summary, these results demonstrate a positive effect of the oxygen carrier M101 on rat and human pancreas during preservation, with an overall improvement in post‐isolation islet quality.

## 1 INTRODUCTION

Pancreatic grafts, using whole organ or islets of Langerhans transplants, can be used to reverse brittle type 1 diabetes and replace dysfunctional insulin‐secreting cells of patients. In the United States, 1002 pancreases were used in whole organ transplantation in 2017, while 2558 people remained on a waiting list.^1^ In islet transplantation, 3‐4 pancreas donors are needed to reverse type 1 diabetes for one recipient.^2^ In 2016, 25% of potentially transplantable pancreases were rejected because of low quality,^3^ which greatly contributed to organ shortage. Pancreas quality depends on the cause of death, age and BMI of the donor, donor medical record and duration of cold ischaemia time.^4^ Between pancreas harvesting and transplantation or islet isolation, a period of storage at 4°C occurs, the only step at which intervention to improve pancreas quality is possible. A longer cold ischaemia time is known to have a negative impact on islet yield^5^ and on transplantation outcome for organs with high metabolic rates.^6^

During ischaemia, a lack of oxygen and nutrients leads to a decrease in high energy phosphate production by mitochondria.^7^ To prevent catabolism related to ischaemia, organs are stored at 4°C. However, because ATP is required for a basal level of metabolism,^8^ the ability of an organ to maintain acceptable levels of adenine nucleotides (ATP, ADP, AMP) during preservation directly relates to its function after transplantation.^9^ In addition to causing a decrease in energy, the degradation of ATP/ADP/AMP into inosine and hypoxanthine/xanthine generates reactive oxygen species (ROS). This oxidative stress can then trigger inflammation through the mitogen‐activated protein kinase (MAPK) pathway and through necrosis.^10^, ^11^ Moreover, a decrease in adenine nucleotide ratios has been reported to correlate with liver dysfunction post‐transplantation.^12^For pancreatic tissue and islets, in particular, the ADP/ATP ratio is negatively correlated with human and porcine islet cell viability, necrosis, apoptosis and function.^13^, ^14^ The detrimental effects of a low energy supply are intensified at the moment of organ isolation (as in reperfusion), generating a large amount of oxidative stress, which has an irreversible effect on cells.^15^ To preserve the pool of adenine nucleotides, one strategy employed in the last decade has focused on increasing oxygen supply, thus ensuring tissue preservation, even at low temperatures.

Various methods to improve oxygenation have been tested during pancreas preservation at low temperatures. Persufflation (gaseous oxygen perfusion) is effective for oxygenating the pancreas, resulting in a decrease in inflammation and increase in human islet metabolic markers.^16^ However, this technique requires specific organ transport materials that are space‐consuming and not cost‐effective. Alternatively, an equipment‐free, two‐layer method (TLM) method has been thoroughly studied, but clinical trials have failed to obtain promising results in clinical settings.^17^, ^18^ TLM uses perfluorocarbons (PFC) as an effective oxygen delivery solution, but a major limitation relates to the fact that PFCs equilibrate quickly with the surrounding atmosphere and therefore lose oxygenation capacity.

A more efficient oxygen transporter is necessary as an alternative to PFCs. Recently, an extracellular haemoglobin called M101 (produced by HEMARINA, Morlaix) isolated from the lugworm *Arenicola marina*, has been developed under the product name of HEMO_2_ life^®^ as an additive to preservation solutions during hypothermic storage of grafts. M101 possesses a high affinity for oxygen and can carry up to 156 oxygen molecules, compared to the four oxygen molecules carried by human haemoglobin.^19^ Oxygen release occurs over a gradient without any allosteric effectors, and according to the p50 of the molecule.^20^ The p50 reflects haemoglobin affinity for oxygen, and the value corresponds to the oxygen tension at which 50% of the O_2_‐binding sites are saturated. This is a crucial advantage of M101 over other types of haemoglobin‐based oxygen carriers (HBOC). The p50 of M101 is 7 mm Hg. The capacity of the molecule to store oxygen is maintained until the pO_2_ rises above 7 mm Hg, at which point a harsh hypoxic environment causes the passive release of oxygen, thus providing the cell with a sufficient oxygen supply. When tissues consume oxygen present in the preservation solution, the pO_2_ decreases below the p50 of M101, releasing oxygen and freeing up a binding site for an additional oxygen molecule. This process contributes to a cycle of continuous oxygen delivery and consumption by cells.

As an extracellular haemoglobin, M101 possesses intrinsic Cu/Zn‐superoxide dismutase (SOD) activity, which protects against potential damages caused by haem‐protein‐associated ROS or ROS related to ischaemia that are released in the solution. M101 is also active from 4‐37°C, which is a suitable temperature for multiple types of organ preservation. M101 is derived from a marine poikilotherm invertebrate that is not able to control its body temperature and undergoes marked seasonal and external daily temperature fluctuations. Efficacy and safety of M101 in organ preservation has been shown to improve graft function for clinical studies on kidney and face transplantation, and for pre‐clinical studies on the kidney, lung and heart.^21^, ^22^, ^23^ Additionally, its ease of use (the molecule can simply be added to the preservation solution) makes it of interest for cold organ preservation, either under static storage or with machine perfusion.^24^, ^25^

The goal of the present study was to identify the impact of cold ischaemia on pancreatic islets through examination of the efficacy of M101 on pancreas cold preservation, using both rat and human models.

## 2 MATERIALS AND METHODS

### 2.1 Antibodies

Primary antibodies against p‐AKT, AKT, p‐ERK1/2, ERK1/2, p‐p38, p38 (anti‐rabbit, 1:1000, Cell Signaling, Ozyme), β‐actin (anti‐rabbit, 1:10,000, Abcam) and HIF1‐α (antimouse, 1:100, Novus Biologicals) were used. Anti‐rabbit and antimouse HRP‐conjugated secondary antibodies were purchased from Sigma‐Aldrich (A0545 and A9044).

### 2.2 Human pancreas and islets

The use of human tissues in the study was approved by the French regulation body (Biomedicine agency, Authorisation number PFS12‐013). For experiments on pancreatic tissue, pancreases were preserved with M101 for the last 3 hours of cold ischaemia (CI) (CI = 9 ± 0 hour; age = 53 ± 7.5 years; BMI = 29.7 ± 7) or without M101 (CI = 9.048 ± 0.29 hours; age = 71 ± 4 years; BMI = 26 ± 1.2). Similarly, for experiments on islets, pancreases were preserved with M101 for last the 3 hours of CI (CI = 9 ± 0 hours; age = 53 ± 7.5 years; BMI = 29.7 ± 7) or without M101 (CI = 8.8 ± 1.34 hours; age = 59.7 ± 12 years; BMI = 29 ± 4.1).

### 2.3 Animals

Wistar rats (male; 250 g; 8‐week‐old) were purchased from Janvier. Animals were housed in standard collective cages under pathogen‐free conditions, in a temperature‐controlled room. They were given the SAFE‐A04 diet, and food and water were available ad libitum. All experiments were performed according to the National Institutes of Health and local ethical committee (CREMEAS) guidelines (Authorisation number: C67‐482‐28).

### 2.4 Experimental design

Rats were anesthetized by intra‐peritoneal injection of IMALGENE^®^ 1000 (Sanofi), a ketamine‐based product, and ROMPUN™ (Sanofi), a muscle relaxant. After laparotomy, 2 mL of preservation solution (CTL: Hanks Balanced Salt Solution (HBSS) solution supplemented with 2.3 mmol/L chloride calcium, 25 mmol/L GIBCO^®^ HEPES, 4.2 mmol/L sodium bicarbonate; Sigma‐Aldrich) was injected into the pancreas via the pancreatic duct. HBSS was chosen as preservation solution since UW solution impaired the islet isolation yielding in our conditions. The pancreas was removed and placed in the preservation solution at 4°C, and cold ischaemia kinetics were then determined (samples were taken at 0, 2, 4, 6, 8, 12 or 18 hours; [n = 4‐6]). The condition we call M101 corresponds to preservation solutions containing M101 (HEMO_2_life^®^, Hemarina) at 2 g/L. Metabolite extraction was performed on fresh tissue, while protein extraction and OCT slide analysis were performed on snap‐frozen tissue at each time‐point. Islet isolation was performed after cold ischaemia (30 minutes, 4, 6, 8, 12 or 18 hours). For the experiments with M101 perfusion, 2 mL of preservation solution with or without M101 at 2 g/L was injected into the pancreas via the pancreatic duct. Pancreases were preserved for 6 hours at 4°C in the presence of M101, before islet isolation process.

Human pancreases (n = 3) underwent 6 hours of cold ischaemia between removal from donors and arrival in our laboratory. M101 (2 g/L) was added to Belzer^®^ solution (UW solution, Bridge to Life) and then injected into the pancreas for 3 hours. Protein extraction was performed on snap‐frozen pancreatic tissue after 1 or 3 hours of M101 exposure at 4°C. Islets were isolated after 9 hours of cold ischaemia and counted after 12 hours culture post‐isolation in CMRL medium (Sigma‐Aldrich) at 37°C. Functionality of islets was assessed by a glucose‐stimulated insulin secretion test.

### 2.5 Preservation solution analysis

Proteins, belonging to the high‐mobility group box 1 (HMGB1) family, are DNA‐binding proteins that regulate transcription. A signal of necrosis 26 occurs when these proteins are released into the extracellular compartment as danger‐associated molecular pattern molecules (DAMPs).^27^ Levels of HMGB1 (ng/mL) and glucose (mmol/L) were quantified in preservation solution after 6 hours of pancreas cold preservation by a HMGB1 ELISA kit (ST51011, IBL International) and glucose RTU assay (BioMérieux), following the manufacturer instructions.

### 2.6 Protein extraction

The total protein content of the frozen pancreatic tissue was extracted using lysis buffer (20 mmol/L Tris, 1 mmol/L EDTA, 1 mmol/L EGTA, 150 mmol/L NaCl, 0.5% Triton X‐100; Sigma‐Aldrich) with HALT™ protease and phosphatase inhibitor cocktail (Thermo Fisher Scientific) and preserved at −80°C. Protein concentration was determined using the bicinchoninic acid assay (BCA; Thermo Fisher Scientific).

### 2.7 Protein analysis

Protein extracts were analysed with the following kits according to manufacturer instructions: mitochondrial complex 1, p38α activity (pT180/Y182) and AKT activity (AKT1/2/3 (pS473) + AKT1 total) were quantified with ELISA kits (Abcam). For mitochondrial complex 1, results were expressed in OD (optical density)/min; for p38α activity (pT180/Y182), results were expressed as the ratio of phosphorylated p38α over total p38α for pancreatic tissue; for AKT activity, results were expressed as the ratio of phosphorylated AKT1/2/3 over total AKT1, and as fold‐increase or fold‐decrease in relation to the control. Active caspase‐3 was quantified by human active caspase‐3 ELISA (R&D Systems), and results were expressed as fold‐increase or fold‐decrease in relation to the control. HIF1‐α expression was measured by human HIF1‐α ELISA (Active Motif), and results were expressed as fold‐decrease in relation to the control.

### 2.8 Western blot

Equal protein amounts from each sample were mixed with sample buffer (Bio‐Rad) and β‐mercaptoethanol (1:100 dilution, Sigma‐Aldrich). Samples were heated at 95°C for 5 minutes, and loaded and separated by electrophoresis on polyacrylamide gel (Criterion XT 4%‐12% Bis‐Tris, Bio‐Rad). Semi‐dry transfer was used for protein transfer on nitrocellulose membranes (Trans‐Blot^®^ Turbo™ Transfer System, Bio‐Rad). Membranes were blocked for 1 hour at 37°C in TBS‐T 5% bovine serum albumin (Sigma‐Aldrich) and were probed with primary antibodies overnight at 4°C. Blots were revealed by SuperSignal™ West Femto Maximum Sensitivity Substrate (Thermo Fisher Scientific) and recorded with a Chemidoc^®^ instrument (Bio‐Rad). Densitometry analysis was performed with Bio‐Rad software, and results were expressed relative to β‐actin.

### 2.9 Metabolite extraction

The total metabolite content of fresh pancreatic tissue was extracted using perchloric acid (PCA 2 mol/L; Thermo Fisher Scientific) and potassium hydroxide (2 mol/L KOH) according to manufacturer protocol and was preserved at −80°C. The metabolite content was normalized according to the weight (mg) of pancreatic tissue used in the extraction (metabolites/µg protein).

ATP, X/H and lactate amount were quantified by ATP assay kit (ab83355, Abcam), xanthine/hypoxanthine assay kit (ab155900, Abcam) and l‐lactate assay kit (ab65331, Abcam), respectively, using the metabolite extract, and following manufacturer instructions. Results were expressed in metabolites/µg proteins or rationalized with the t0h values corresponding to the time point 0h.

### 2.10 Histological studies

Pancreases were either fixed and frozen in OCT, or fixed in paraformaldehyde. Sections were immunostained with antibodies against HIF1‐α, and slides were then mounted with a media containing 1.5 µg/mL of 2‐(4‐amidinophenyl)‐1H‐indole‐6‐carboxamidine (DAPI; FluorSave Reagent, Merck Millipore) and observed using a Nikon Eclipse 50i microscope and Nis‐Elements‐BR software. HIF staining was quantified on five sections of each pancreas using Nis‐Elements‐Br software. Results are expressed as intensity of staining relative to the total surface area.

For ROS staining, pancreases were sectioned using a cryostat, distributed onto glass slides and preserved at −80°C. Total ROS level was assessed by dihydroethidium (DHE) staining for 30 minutes. Fluorescence was quantified using Nis‐Elements‐Br software, and results were expressed as a ratio with respect to the control.

### 2.11 Islet isolation, functionality, counting and viability

Pancreatic islets were isolated using standard collagenase (Sigma‐Aldrich) digestion and Ficoll (Eurobio, Les Ulis, France) purification. Rat islets were cultured in Medium 199 (Thermo Fisher Scientific), while human islets were cultured in CMRL media (Sigma‐Aldrich). Both media were supplemented with 10% heat‐inactivated foetal bovine serum (Sigma‐Aldrich) and 1% Amphotericin B/ Penicillin/ Streptomycin (Gibco, Life Technologies). A subset of freshly isolated islets from each experimental condition were washed extensively and incubated in 10% BSF‐complemented Krebs‐Ringer bicarbonate medium for 1 hour at 37°C with 2.8 mmol/L glucose under basal conditions and 16.7 mmol/L glucose under stimulatory conditions. Supernatants were frozen, and insulin measurements were performed using a rat or human insulin ELISA kit (Mercodia). Supernatant insulin content was expressed in µg/L per 10 islets, and the stimulation index (SI) was defined as the ratio of insulin secreted under stimulated conditions over the mean that secreted under basal conditions. Islets were counted using islet equivalent measurements (IEQ), using the consensus reported by Ricordi 28 that one IEQ is equal to a pancreatic islet with a diameter of 150 μm.

### 2.12 Statistical analysis

Statistical analyses were performed using Statistica software (StatSoft, Maisons‐Alfort). Results were analysed by aKruskall‐Wallis and Wilcoxon/Mann‐Whitney test. Results are expressed as mean ± SEM, or as scattered plots showing the median.

## 3 RESULTS

### 3.1 Cold ischaemia kinetics in pancreatic tissue

#### 3.1.1 Rat pancreatic tissue

**Figure 1:**
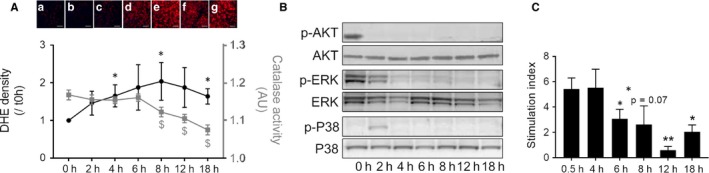
Impact of cold ischaemia kinetics on Rat pancreatic tissues. A, reactive oxygen species (ROS) staining of 5 µmol/L sections and representative images. Red staining represents ROS levels in rat pancreatic tissue (n = 5). Catalase activity was measured by ELISA, and results are expressed as arbitrary units (n = 6). B, AKT, ERK and p38 activation (phosphorylated protein and total protein) were analysed by Western blot. Blots are representative of three independent experiments. C, Stimulation index of rat islets was assessed after different cold ischaemia times (30 min, 4, 6, 8, 12 or 18 h). *** *P* < .001; ** *P* < .01; * *P* < .05. Results are expressed as mean ± SEM

**Figure 2:**
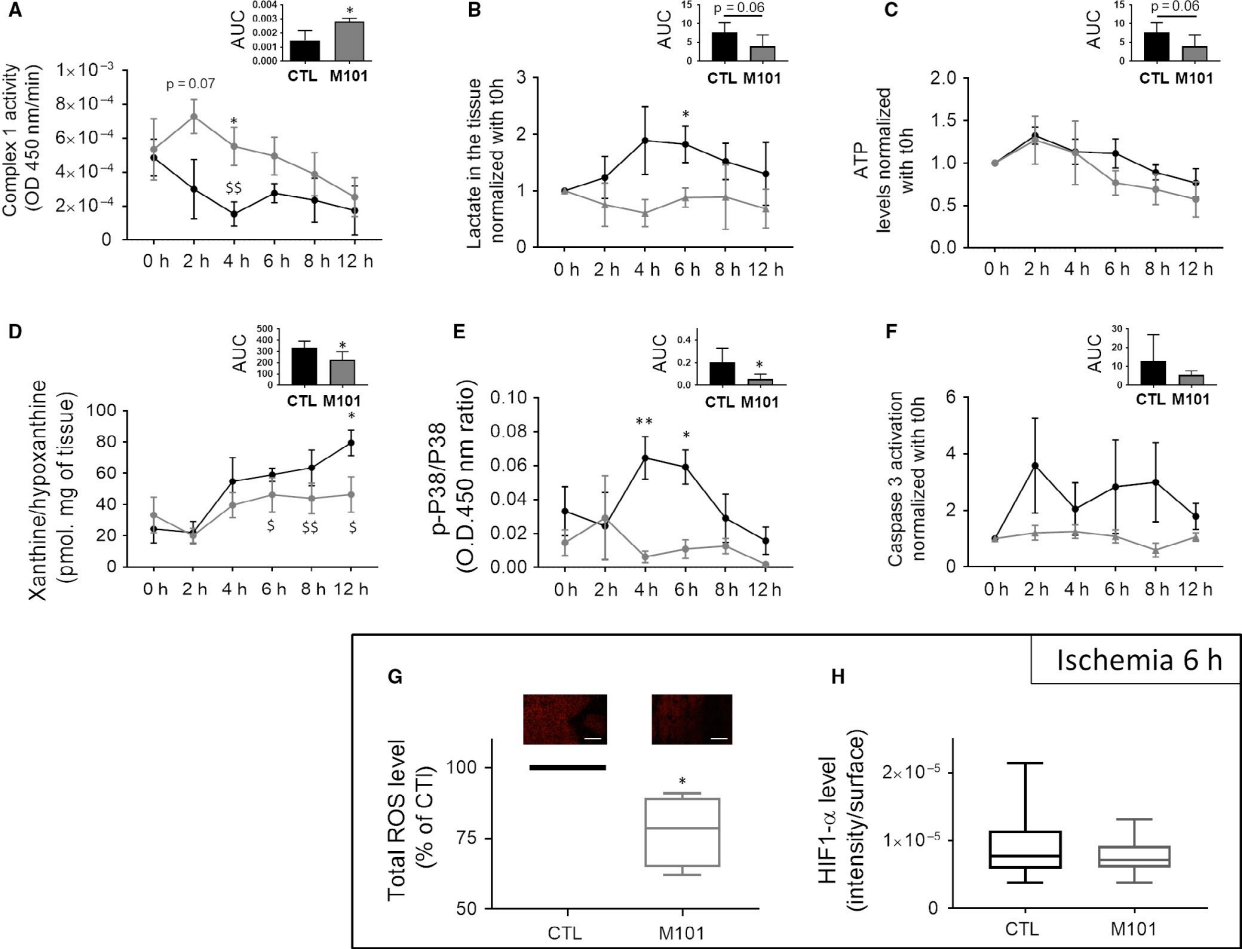
Ischaemia markers in Rat pancreases modulated by the presence of M101 in the preservation solution (2 g/L). A, Mitochondrial complex 1 activity was assessed in pancreatic tissue (n = 4). B, Lactate level (normalized with t0h) (n = 4), (C) ATP and (D) xanthine/hypoxanthine (X/H) level (pmol.mg of tissue) were measured (n = 6). E, p38 activation (phosphorylated protein/total protein) was analysed by ELISA (n = 4). F, Cleaved caspase‐3 levels (normalized with t0h) were assessed in pancreatic tissue (n = 6). G, reactive oxygen species (ROS) staining of 5 µmol/L sections and representative images. Red staining represents ROS levels in rat pancreatic tissue (n = 4). H, HIF1‐α level (fluorescence intensity/surface ratio) was assessed by immunostaining of frozen slides of pancreatic tissue (n = 4). Significant differences are represented with * for comparisons of CTL and M101, and with $ for comparison between t0h and other time‐points for the CTL condition. ** *P* < .01; * *P* < .05. Results are expressed as mean ± SEM

Various markers of pancreas metabolism related to respiration, stress or defence were studied. The modulation of each marker was time‐dependent, highlighting the phases of ischaemic effects on tissue. The level of total ROS increased with time of cold ischaemia (*P* = .0286), while catalase activity was maintained at constant levels until after 6 hours of ischaemia, before a decrease in activity from 8 hours (*P* = .041) to 18 hours (*P* = .002; Figure 1A). Ischaemia induced a rapid inhibition of AKT phosphorylation after 2 hours (Figure 1B). Similarly, ERK phosphorylation was decreased with time of ischaemia (Figure 1B). A transient increase in p38 phosphorylation (Figure 1B and Figure 2E) was observed after 2‐4 hours of ischaemia, before a loss of phosphorylation at the longer time‐points (8 hours Figure 2E). Mitochondrial complex 1 activity was decreased after 2 hours of ischaemia (Figure 2A, *P* = .029). Levels of lactate in the pancreas increased for the first 4 hours and then decreased slowly at longer times of ischaemia (Figure 2B). Preservation of the pancreas at 4°C resulted in stabilization of ATP concentrations during short ischaemia (0‐6 hours) and a decrease for longer ischaemia (Figure 2C, *P* = .041). The level of xanthine/hypoxanthine, a degradation product of ATP, was increased during long ischaemia times (8 hours (*P* = .0152), 12 hours (*P* = .004) or 18 hours (*P* = .002)) in pancreatic tissue (Figure 2D). Caspase‐3 activation was not modified during ischaemia (Figure 2F).

#### 3.1.2 Rat pancreatic islets

The endocrine portion of the pancreas is the most sensitive to ischaemia, and islet quality can serve as a barometer to measure irreversible impacts of ischaemia. About 4 hours of ischaemia had no impact on islet yield, but longer times (8 hours (*P* = .045), 12 hours (*P* = .001) or 18 hours (*P* = .0009) drastically decreased the islet yield per pancreas (Figure S1). When staining with FDA/PI was performed (Figure S1), we observed a decrease in islet viability after 18 hours of ischaemia (*P* = .019). (Figure 1C).A glucose‐stimulated insulin secretion test (Figure 1C) demonstrated a decrease in islet functionality (with Stimulation index representing islet response to glucose stimulation) after 6 hours of ischaemia (*P* < .05) and at longer CIT (*P* = .013 and *P* = .018, at 12 and 18 hours, respectively).

### 3.2 Biomarker evolution over 12 hours of rat pancreas preservation in the presence of M101

The presence of M101 triggered the maintenance of mitochondrial complex 1 pancreas activity throughout ischaemia kinetics (Figure 2A), as compared to the control (AUC, *P* = .027). Lactate levels in the presence of M101 were maintained at a lower level than the control (Figure 2B). No differences in ATP levels were observed with M101 (Figure 2C), while global levels of X/H throughout the experiment were lower in the presence of M101 (AUC, *P* = .0152; Figure 2D). M101 suppresses transient phosphorylation of p38, which is observed in the control, after 4 hours (*P* = .002) and 6 hours (*P* = .008) of ischaemia (Figure 2E). M101 also reduced variability of active caspase‐3 levels compared to that in the control (Figure 2F).

Dihydroethidium staining showed a 25% decrease in total ROS levels in the presence of M101, as compared to a control, after 6 hours of ischaemia(*P* = .027)(Figure 2G). Immunostaining revealed no differences in HIF1‐α levels in tissue after 6 hours of ischaemia (Figure 2H).

**Figure 3:**
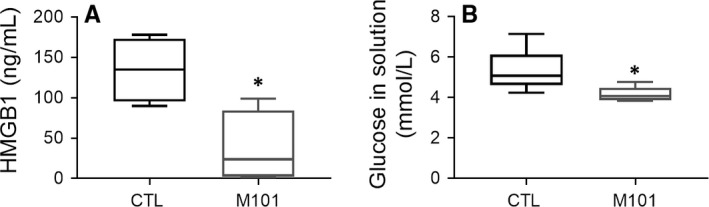
A decrease in necrosis and increase in glucose consumption was observed in Rat pancreatic tissue preserved with 2 g/L of M101. A, HMGB1 quantity (ng/mL) released by the pancreas was measured in the preservation solution by ELISA after 6 h of CI. B, Glucose quantity (mmol/L) was assessed in the preservation solution after 6 h of CI. Results are expressed as mean ± SEM of four independent experiments

A decrease in HMGB1, a marker of necrosis/inflammation, released by pancreatic cells, was observed with M101 treatment as compared to the control after 6 hours of ischaemia (Figure 3A; *P* < .05).Glucose levels within the preservation solution, reflecting glucose consumption and therefore cellular metabolism, were decreased after 6 hours compared to the control (*P* = .0159; Figure 3B).

### 3.3 M101 protects rat islets from ischaemia through pancreas preservation

**Figure 4:**
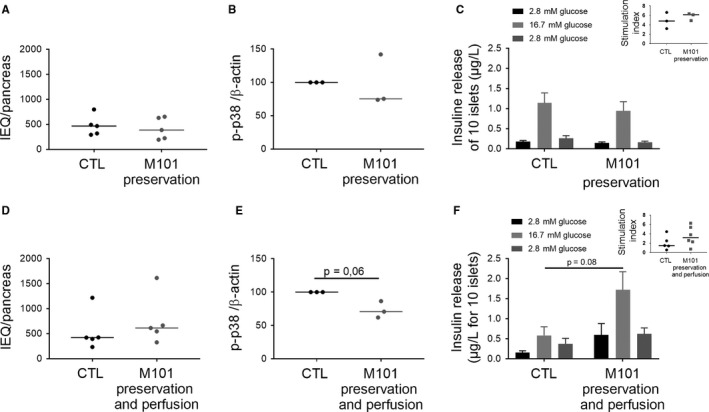
Rat islet quality was improved with M101 use for pancreas preservation when injected in the organ. M101 was added in the preservation solution (A, B, C, D, E, F) and injected in the pancreas (D, E, F). A, D, Islets were counted using Islets Equivalents (IEQ) just after isolation (n = 5). B, E, p38 activation (phosphorylated protein/β‐actin ratio) was analysed by Western blot in islets (n = 3). C, F, Amount of insulin secreted by islets under glucose stimulation was quantified by ELISA (n = 5). ** *P* < .01; * *P* < .05. Results are expressed as mean ± SEM or median

Islet quality was assessed immediately after isolation, and after 6 hours of cold ischaemia in the presence of M101, combined with M101 injection via the duct. Insulin secretion in response to glucose tended to be higher as compared to control conditions (*P* = .08; Figure 4F).M101 injection also reduced p38 activity in islets (*P* = .0648; Figure 4E), and no impact of M101 injection was observed on stimulation index nor islet yield (Figure 4D). When M101 was present in the preservation solution only, no influence of the molecule was observed on the parameters tested (islet yielding (Figure 4A), p38 activation (Figure 4B) or insulin secretion (Figure 4C)).

### 3.4 M101 protection of human pancreases and islets

**Figure 5:**
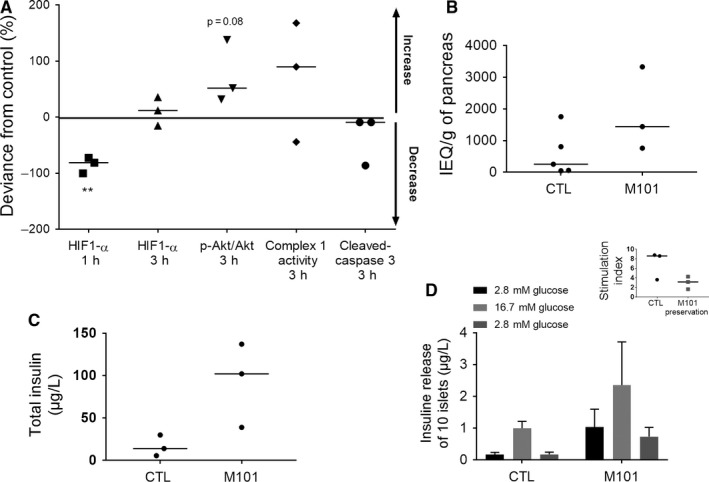
Human pancreas and human islet preservation are improved by the use of M101 for pancreas preservation. M101 was injected into the pancreas and in the preservation solution for 3 h. The impact of the molecule was observed both on the pancreatic tissue and islets after isolation. A, HIF1‐α level was assessed by ELISA in pancreatic tissue after 1 h and 3 h of cold ischaemia. AKT activation (phosphorylated protein/total protein ratio), mitochondrial complex 1 activity, and cleaved caspase‐3 levels were assessed in pancreatic tissue by ELISA after 3 h of cold ischaemia. B, Islet isolation was performed, and islets were counted using Islets Equivalents (IEQ) after one night of culture at 37°C. C, The total amount of insulin in islets and (D) insulin secretion of islets under glucose stimulation was quantified by ELISA. Results are expressed as mean ± SEM or median. (M101 treatment n = 3; CTL n = 5)

M101 contributed to a decrease in HIF1‐α expression in pancreas after 1 hours of exposure, but this effect was not present after 3 hours (Figure 5A). AKT phosphorylation in tissue increased after 3 hours of exposure to M101 (*P* = .08; Figure 5A). Two out of three pancreases had increased complex 1 activity, and all the three had less cleaved caspase‐3 in tissue, but this difference was not significant (Figure 5A). A Spearman's correlation was performed to check the influence of age on IEQ or insulin secretion. Despite the low number of islet isolations, we observed that islet yield (Figure 5B) and function of M101‐treated pancreases improved relative to the control. Islets seem to be able to secrete higher amounts of insulin both in basal and in stimulated conditions and a possible decrease in stimulation index (Figure 5D). Islets from M101‐preserved pancreases also showed an overall increase in insulin content (Figure 5C).

## 4 DISCUSSION

The goal of this study was to evaluate the impact of the oxygen carrier M101 during preservation of rat and human pancreatic tissues exposed to cold ischaemia. The use of M101 resulted in a delay in ischaemia impact on rat pancreatic tissues, maintenance of oxidative phosphorylation (marked by a decrease in lactate production and maintenance of mitochondrial activity), and a reduction in oxidative stress and necrosis during cold ischaemia. M101 also improved islet glucose‐stimulated insulin secretion and had beneficial effects on human pancreatic tissues and islets.

Time spent in cold ischaemia is known to have a greater impact on both exocrine and endocrine pancreatic tissue than the process of cold ischaemia itself.^29^ Cold ischaemia time is negatively correlated with islet yield, islet function in vitro^5^ or post‐transplantation,^30^ revealing that preservation at 4°C is not sufficient for optimal preservation Despite the fact that a preservation solution could not be used in our conditions, we confirmed those observations using HBSS in the present study. Although cellular ATP dependence is reduced by the cold temperature (a metabolism/catabolism reduction), cells still need a certain amount of ATP to maintain basal metabolism levels. The production of ATP is dependent primarily on oxygen availability. When an organ is disconnected from the blood supply, oxygen access is limited to diffusion against a gradient. By consuming oxygen in the preservation solution, cells decrease the oxygen concentration in the medium, which is in direct contact with tissue. This local decrease in oxygen is then counterbalanced by oxygen diffusion through the solution,^31^ which is dependent on the amount of dissolved oxygen in the solution, as well as cellular consumption.^32^ The oxygen concentration decreases from 20% at the air/solution interface to nearly 0% at regions close to the tissue (data not shown) with a continuous gradient created by cell consumption. Furthermore, this gradient is important for oxygen penetration in tissues, which occurs to a depth of only 5 mm, as a greater amount of oxygen in the solution allows for deeper tissue penetration. In a typical situation, almost no oxygen is in direct contact with the tissue, triggering a reduced respiration rate and glycolysis in the presence of glucose.^33^, ^34^ To survive, cells switch to anaerobic metabolism to produce a small amount of ATP by glycolysis, which leads to poor yields, with only two ATP molecules per glucose vs 36 ATP produced under aerobic condition.^7^ In this study, this phenomenon was demonstrated by a mitochondrial complex 1 activity decrease, leading to low ATP levels in the pancreatic tissue. Lactate accumulation in the tissue after 4 hours of cold ischaemia shows that glycolysis was triggered in order for the tissue to survive cold ischaemia. At 4 hours post‐retrieval, oxidative stress concomitant to the activation of MAPK p38 is initiated, marking the beginning of cell stress. p38 is known to be involved in responding to cellular stressors such as hypoxia, which can trigger inflammation and eventually cell death.^35^ Furthermore, the central role of p38 during ischaemia was shown by reduction in infarct size when p38 was specifically inhibited.^36^ After 6 hours, a further decrease in ATP concomitant with a xanthine/hypoxanthine increase revealed that cells are unable to cope with a longer ischaemia time.^37^ Additionally, it is known that ROS production induced by mitochondrial dysfunction leads to structural and functional cell damages.^38^ Two distinct phases of cold ischaemia were observed up until 6 hours in this study, and our results confirm observations in several other organs, such as the liver and kidney.^39^, ^40^, ^41^ We confirmed that beyond 6 hours, islets started to lose function. However, we cannot exclude that this effect might be due to the use of HBSS and further studies using preservation solution would strengthen the study. Nevertheless, this observation is supported by different studies performed on human islets from pancreases and preserved mainly in UW stating that it exists a correlation between the cold ischaemia time and the decrease in islet function.^42^, ^43^, ^44^, ^45^

Addressing oxygenation and oxidative stress is the best means to improve organ quality, as has already been demonstrated in various tissues.^46^, ^47^, ^48^ M101 helps to maintain oxidative phosphorylation in the tissue during the first few hours of cold ischaemia, as shown by the maintenance of mitochondrial complex 1 activity. This result demonstrates the ability of cells to use M101‐released oxygen present in the medium, in a gradient‐dependent manner. The oxygen supply of the preservation solution is important, and maintenance of the gradient is related to oxygen release by M101. Interestingly, the maintenance of cell respiration in the presence of M101 was not correlated with an increase in ATP, despite increased glucose uptake, which has not been previously shown in the literature.^49^, ^50^, ^51^ However, a decrease in xanthine/hypoxanthine production (a degradation product of ATP) shows that the ATP pool was less degraded^37^ as compared to the control. Further experiments are necessary to completely understand this effect.

After 6 hours of cold ischaemia in the presence of M101, a 25% decrease in ROS production in the pancreas was found when compared to the control, which could be due to the intrinsic SOD‐like activity of M101. Radical production leads to an exponential chain reaction and damages related to oxidative stress can lead to necrosis, which can be observed with specific markers such as HMGB1. HMGB1 is known to be released by oxidative stress‐induced necrotic cells.^27^, ^52^, ^53^ To break this chain reaction and prevent spreading of oxidative stress, ROS must be converted to harmless molecules by antioxidant enzymes or scavenger receptors. We observed this effect in the presence of M101, where intrinsic SOD‐like activity led to a decrease in ROS production and a subsequent decrease in necrosis (HMGB1). Thus, a cycle was initiated by M101, in which a decrease in hypoxia first occurred, followed by decrease in oxidative stress by SOD through the harvesting and recycling of ROS, resulting in an overall decrease in tissue inflammation and necrosis.

M101, when present in both the preservation and perfusion solutions, also improved islet function and led to an increase in insulin secretion in response to glucose stimulation (data not shown for an increase in GLUT2 expression) concomitantly with a decrease in p38‐MAPK pathway activity. When M101 was not injected and only present in the preservation solution, no impact was observed on islet quality. Islets were not exposed directly to the molecule and thus did not benefit from oxygenation and antioxidant activity. Injection via the duct potentiated the beneficial effect of M101 and was further improved by direct administration via the vein so that the molecule was delivered into the core of the islets. This mode of administration would also be greatly beneficial for endothelial cells, which are very sensitive to the deleterious events of ischaemia.^54^, ^55^

Our rat model results were confirmed on human pancreas, even though differences between rat and human tissue thickness and the difference in preservation solutions could have influenced the effect of M101.^56^ For logistical reasons, addition of M101 to human pancreas preservation solution was performed only after 6 hours of cold ischaemia. Despite the absence of M101 during this first period of ischaemia, positive effects for M101 were observed. After 1 hours of exposure, HIF1‐α expression in pancreases decreased, and after 3 hours, M101 improved cell survival, leading to an increase in AKT activation and decrease in cleaved caspase‐3. These results confirm the positive impact that we observed in rat tissue. Despite the transient efficacy of M101 on oxygenation in pancreatic tissue, the antioxidant effect protected tissues, resulting in an islet yield increase when compared to non‐treated pancreases. Islets also had a higher insulin content, which was correlated with a greater secretion of insulin by islets under both basal and stimulated conditions. This result is of interest in cases of islet transplantations.

In summary, we observed beneficial effects of M101 on rat tissue and confirmed these results in a sample of human pancreases, thus showing that the use of M101 both in the preservation and perfusion solutions offers protection during cold ischaemia and will improve transplantation outcome.

## CONFLICT OF INTEREST

The authors declare that F. Z. is the founder of HEMARINA SA and holds stock in the company, which produces the substance under investigation. E. D. is an employee of HEMARINA SA and does not hold stock.

## AUTHOR CONTRIBUTIONS

FL and EM designed and performed the research and wrote the paper. SS, FZ, ED, KB and MP gave critical advice on the paper writing. FL, JC and CP performed technical experiments.

## Data Availability

The data that support the findings of this study are available from the corresponding author, EM, upon reasonable request.
