# Supplementary figures and images for: Beneficial effects of the novel marine oxygen carrier M101 during cold preservation of rat and human pancreas
Source: J Cell Mol Med. 2019 Oct 11;23(12):8025–34. doi: 10.1111/jcmm.14666 (PMC6850937; doi:10.1111/jcmm.14666)

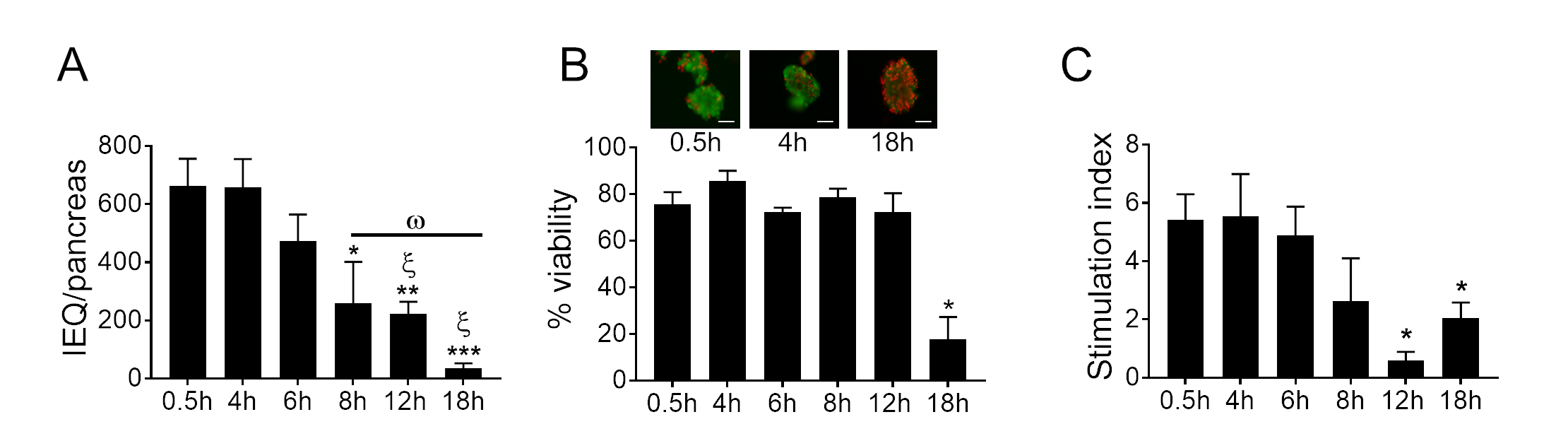

Supplement: Supplementary file 1 [file JCMM-23-8025-s001.tif]
